# Oral mucositis in patients with acute myeloid leukemia treated with allogeneic hematopoietic stem cell transplantation in relation to the conditioning used prior to transplantation

**DOI:** 10.1007/s00277-021-04568-y

**Published:** 2021-06-12

**Authors:** Aleksandra Wysocka-Słowik, Lidia Gil, Zuzanna Ślebioda, Agnieszka Kręgielczak, Barbara Dorocka-Bobkowska

**Affiliations:** 1 Department of Gerodontology and Oral Pathology, Poznan University of Medical Sciences, Bukowska 70, 60-812 Poznań, Poland; 2 Department of Hematology and Bone Marrow Transplantation, Poznan University of Medical Sciences, Szamarzewskiego 84, 60-569 Poznań, Poland

**Keywords:** Acute myeloid leukemia, Oral pathology, Oral mucositis, Myeloablative conditioning, Reduced intensity conditioning

## Abstract

This study was designed to investigate the frequency and severity of oral mucositis in patients with acute myeloid leukemia after allogeneic hematopoietic cell transplantation, in relation to the type of conditioning used. Eighty patients diagnosed with acute myeloid leukemia were assigned to two groups based on the conditioning regimen used before transplantation. The intensity of oral inflammatory lesions induced by chemotherapy (oral mucositis) was evaluated according to a 5-point scale recommended by World Health Organization. Oral mucosa was investigated in all patients before the transplantation and during two subsequent stages of the post-transplantation procedure in relation to the conditioning regimen used. Mucositis in the oral cavity was observed in the majority of patients (66%) in the first week after transplantation, whereas the largest percentage of patients suffering oral lesions (74%) occurred in the second week after transplantation. A significantly higher percentage of patients with mucositis was observed in the group which underwent myeloablation therapy (74% of MAC and 50% of RIC patients in the first week; 83% of MAC and 53% of RIC patients in the second examination).The severity of mucositis after transplantation was higher in the MAC patients compared to the RIC patients. The highest mean value of the mucositis index was recorded in the second week in the MAC group (1.59). In AML sufferers receiving allo-HSCT, oral mucositis is a significant complication of the transplantation. This condition is more frequent and more severe in patients after treatment with myeloablation therapy.

## Introduction

Acute myeloid leukemia (AML) is a proliferative disease of the hematopoietic system, characterized by uncontrolled clonal proliferation of neoplastic hematopoietic precursors. This results in a disturbed production of normal blood cells in blood, bone marrow, and other tissues. AML accounts for about 80% of all acute leukemia cases in adults. The disease risk increases with age. The average age of AML patients being 69 years as reported by various authors [1–3]. The treatment options for AML depend on the patient’s age, general health, cytogenetic, and molecular risk. The implementation of a therapeutic approach based on conventional chemotherapy has resulted in a total remission in 60–80% of adults with AML de novo under 60 years of age [4–6]. AML is currently the leading indication for allogeneic hematopoietic stem cell transplantation (allo-HSCT).

Prior to transplantation, patients require high doses of antiproliferative and cytostatic drugs (myeloablative conditioning, MAC). Although the intensive conditioning regimen decreases the risk of relapse after transplantation, it is characterized by high toxicity [7]. Reduced intensity conditioning (RIC), which is an alternative pre-transplant procedure, was designed to suppress the patient’s immune system enough to accept the donor stem cells, while being less toxic than MAC. However, the risk of transplant rejection is higher for this type of procedure.

Oral mucositis is a significant and one of the most common oral complications of high-dose chemotherapy and total body irradiation (TBI) observed in early stage after hematopoietic stem cell transplantation (HCT) [8–10]. It develops as a consequence of the direct action of cytostatics on the oral epithelium, but it is also due to an impaired immune system function and decreased salivation. During anticancer therapy, the regeneration of the damaged epithelial cells is disturbed. That leads to the formation of oral erosions and ulcers, which may become a portal of entry for several viral, fungal, and bacterial antigens [9, 11, 12]. Infections are a common cause of morbidity and mortality in children and adults after hematopoietic cell transplantation (HCT). Due to antibiotic resistance, bacterial infections are associated with high mortality both after allo- and auto-HCTs, while invasive fungal disease remains an important cause of morbidity and mortality particularly after allo-HCT. A high risk of viral infection was mainly observed after allo-HCT, which could be attributed to the delayed immune reconstitution after transplantation [13]. Hematologic deficits, a common complication in AML subjects, also interfere with oral mucosa condition. Iron and vitamin B_12_ deficiencies may cause a reduction in the thickness of the oral epithelium, which makes it more vulnerable, while a decreased accessibility of transferrin-bound iron leads to a disruption of the lymphocytes and pro-inflammatory cytokines production [14].

The advanced stage of mucositis often requires intensive medical care. OM may impact the patients’ food and fluid intake, significantly prolong the duration of hospitalization, and compromise the response to treatment [10].

Because the reduced intensity conditioning enables the treatment of older adults and patients with coexisting systemic diseases, the number of allo-HSCT procedures has been progressively increasing [7]. Therefore, there is a need for a thorough analysis of the health status of patients with AML after allo-HSCT, with particular attention to oral mucositis.

## Materials and methods

The study group consisted of 80 patients (42 women and 38 men), aged 19 to 69 years (mean 46.6 ± 13.6), diagnosed with AML. All patients underwent allogeneic hematopoietic cell transplantation from December 2015 to July 2018.

Depending on the conditioning, the patients were assigned to one of two groups. The first group consisted of 54 patients (30 women and 24 men), with a mean age of 42.3 ± 11.9 years, who underwent myeloablation therapy (MAC). The other group consisted of 26 patients (12 women and 14 men) with a mean age of 55.5 ± 12.9 years, who were treated with reduced intensity therapy (RIC). The qualification of patients into MAC and RIC regimen was performed by hematologist in the Department of Hematology and Bone Marrow Transplantation of PUMS based on the two major criteria: the age of the patient and the presence of concomitant diseases, as evaluated with reference to the hematopoietic cell transplantation-specific comorbidity index (HCT-CI) according to Charlson et al. [15].

Cytostatics used in the MAC group included the following: fludarabine, busulfan, melphalan, and treosulfan, individually adjusted to each patient’s profile and applied in appropriate proportions as FluBu4 (34 patients), FluBu3 (12 patients), and MelFluTreo (8 patients). RIC therapy consisted of fludarabine, busulfan, cytarabine, and total body irradiation (TBI), used as FluBu2 (17 patients) or FluCyTBI (9 patients). Each patient was examined three times according to the following scheme used in the Department of Hematology and Bone Marrow Transplantation of PUMS:

- (A) Preliminary examination — in the period preceding bone marrow transplantation from day − 10 to day − 7.
- (B) First examination — after transplantation of hematopoietic cells from day + 3 to day + 7.
- (C) Second examination — after hematopoietic cell transplantation from day + 8 to day + 14.

The intensity of oral mucositis was evaluated according to 5-point scale recommended by the World Health Organization (WHO) [16, 17] and described below:

- 0 — No signs
- 1 — Oral soreness + / − erythema, no erosions, patient may complaint on oral discomfort
- 2 — Oral erythema, ulcers; solid diet tolerated
- 4 — Diffuse oral erythema, ulcers; liquid diet only
- 5 — Severe inflammation, diffuse inflammatory-necrotic lesions; alimentation not possible

Prevention of oral mucositis in both groups of our study involved the maintenance of good oral hygiene that included brushing with a soft toothbrush 4 times a day and avoiding substances which caused local irritation (alcohol mouth rinses and acidic, salty, or dry foods). A supersaturated calcium phosphate, electrolyte mouth rinse (caphosol, Fomukal) was recommended to all patients to be used 4 times a day and a systemic antifungal drug (fluconazole) was included as a standard procedure. For the treatment of oral mucositis, patients were advised to use multipurpose mouthwashes and antifungals (benzocaine/natrium, boricum/glicerini, thymol/glycerini, and colistin/gentamycin/nystatin, amphotericin B). Morphine and tramadol were utilized in systemic pain management.

The results were statistically analyzed with Statistica.PL ver. 13.0 (StatSoft, Inc., 2014) for Windows with t-Student, Kruskal–Wallis, Mann–Whitney U test, and test of the difference between tests with p < 0.05 considered as a significance level.

## Results

**Fig. 1:**
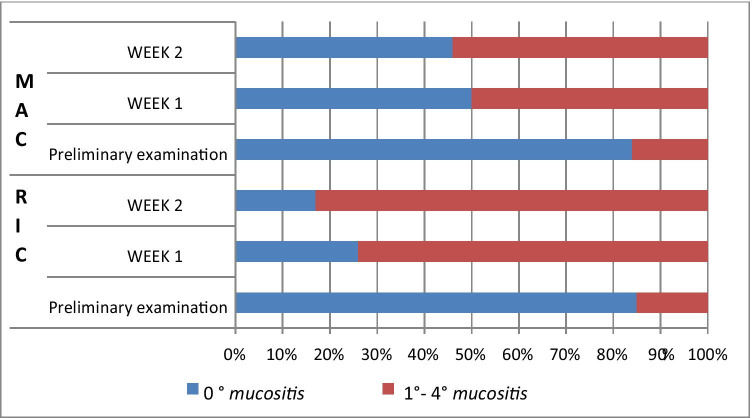
The incidence of mucositis in patients with AML with respect to the type of conditioning (MAC, myeloablative chemotherapy; RIC, reduced intensity chemotherapy)

Based on the physical examination, the intensity of inflammatory changes in the oral mucosa after chemotherapy (oral mucositis) was analyzed according to the five-level classification by the World Health Organization. The changes were assessed subsequent to the transplant procedure, in the entire study population (Table 1) as well as taking into account the type of therapy used in the preparation for allo-HSCT (Table 2). Altogether, within the studied group, 67 patients (84%) showed no symptoms typical of 0° mucositis (according to the WHO criterion) in the preliminary study. In the first post-transplant study, this number decreased to 27 patients (34%) and in the second week to 21 (26%), which represents a statistically significant difference (p < 0.0001) (Table 1). When analyzing the incidence of 0° mucositis in relation to the type of conditioning, no significant differences were found between the two groups in the preliminary examination (46 MAC patients, i.e., 85%, 21 RIC patients, i.e., 84%). In the first week after transplantation, no signs of mucositis were noted in 14 patients (26%) in the MAC group and in 13 patients, i.e., half of the number, in the RIC group (50%). This difference was statistically significant (p = 0.0335). In the second examination, 0° mucositis was recorded in only 9 patients (17%) belonging to the MAC group, compared with 12 patients (47%) within the RIC group, which is a statistically significant difference (p = 0.0059) (Table 2, Fig. 1). Mucosal redness, swelling, and discomfort in the oral cavity, i.e., grade 1 mucositis, were found in the initial examination in 10 patients (12.5%) of the entire study population, in 34 patients (43%) in the first examination, and in 26 (33%) in the second examination (Table 1). The differences between the preliminary and post-transplant examinations were statistically significant (p < 0.0001 and p = 0.0020). During the preliminary examination, in the MAC group, 1° mucositis was identified in 7 patients (13%) and in the RIC group in 3 patients (12%). In the first examination, the incidence of 1° symptoms increased to 46% (25 patients) in the MAC group and to 35% (9 patients) in the RIC group. In the second week, it remained unchanged at 35% in the RIC group, while in the MAC group, the percentage change was 31% (17 patients) (Table 2). The 2° mucositis (appearing as a redness of the oral mucosa) and the presence of erosions (photo 1) was found before transplantation in only 3 subjects, i.e., in 3.5% (1 patient, i.e., 2%, in the MAC group and 2 patients, i.e., 4%, in the RIC group), while in the first week after transplantation, it was found in 10 subjects (19%) in the MAC group and in 2 subjects (8%) in the RIC group (12 patients in total, i.e., 15%). In the second week, the incidence of the 2nd degree lesions increased in both the MAC (18 patients, 33%) and the RIC groups (4 patients, 15%). The percentage of patients with 2° symptoms in the entire study population was statistically significantly lower in the initial examination compared to that in the first and second examinations (p = 0.0121 and p < 0.0001), while the differences between the MAC and RIC groups were not statistically significant. The 3° mucositis (photo 2), with mucosal ulceration and with the patient being able to drink fluids only, was identified in the first examination in 4 (7%) patients in the MAC group and in 1 patient (3.5%) in the RIC group (a total of 5 patients, i.e., 6%). In the second week, the frequency of 3° lesions in the MAC group increased to 15% (8 people), while in the RIC group, it decreased to 0% with the difference between MAC and RIC groups being statistically significant (p = 0.0372). In both groups, no patients were found to have 3° mucositis during the preliminary examination (Table 2, Fig. 1). The differences in the entire study population with respect to the percentage of patients with 3° mucositis between the initial and subsequent examinations after transplantation were statistically significant (p = 0.0261, p = 0.0037). The condition in which the patient cannot be fed orally, with signs of severe inflammation and extensive inflammatory-necrotic changes (4° mucositis), was observed during the post-transplant examinations in a similar proportion of subjects in both groups, i.e., in 2% during the first examination (1 patient) and in 3.5% (1 patient) in RIC, in the second week in 4% in both MAC (2 patients) and RIC (1 patient) groups. A total of 2 patients (2%) in the first examination and 3 patients (3.5%) in the second examination were diagnosed with 4° mucositis. However, no symptoms suggestive of 4° inflammation were observed in any patient during the preliminary examination. However, the differences were not statistically significant (Tables 1 and 2). Moreover, it was shown that in the MAC group, the decrease in the incidence of 0° mucositis was statistically significant in subsequent post-transplant examinations compared to the initial examination (p < 0.0001, p < 0.0001), while there was a statistically significant increase in the incidence of 1° mucositis (p = 0, 0002, p = 0.0240), 2° mucositis (p = 0.0040, p < 0.0001), and 3° mucositis (p = 0.0478, p = 0.0031) in both post-transplant examinations. Within the MAC group, no statistically significant differences were found between consecutive weeks for the incidence of 4° mucositis. In the RIC group, significant differences appeared only between the initial examination and the first week (p = 0.0091), and between the initial examination and the second examination (p = 0.0041) for 0° mucositis (Table 2, Fig. 1).

**Table 1:** The incidence of mucositis in the entire study population

Mucositis grade	Total					
	Preliminary examination		Week 1		Week 2	
	n	%	n	%	n	%
0	67	84	27	34	21	26
1	10	12.5	34	43	26	33
2	3	3.5	12	15	22	27.5
3	0	0	5	6	8	10
4	0	0	2	2	3	3.5

**Table 2:** The incidence of mucositis in patients with AML depending on the type of conditioning

Mucositis grade	MAC (N = 54)						RIC (N = 26)					
	Preliminary examination		Week 1		Week 2		Preliminary examination		Week 1		Week 2	
	n	%	n	%	n	%	n	%	N	%	n	%
0	46	85	14	26	9	17	22	84	13	50	12	46
1	7	13	25	46	17	31	3	12	9	35	9	35
2	1	2	10	19	18	33	2	4	2	8	4	15
3	0	0	4	7	8	15	0	0	1	3.5	0	0
4	0	0	1	2	2	4	0	0	1	3.5	1	4

(*MAC*, patients after myeloablative chemotherapy; *RIC*, patients after reduced intensity chemotherapy)

**Fig. 2:**
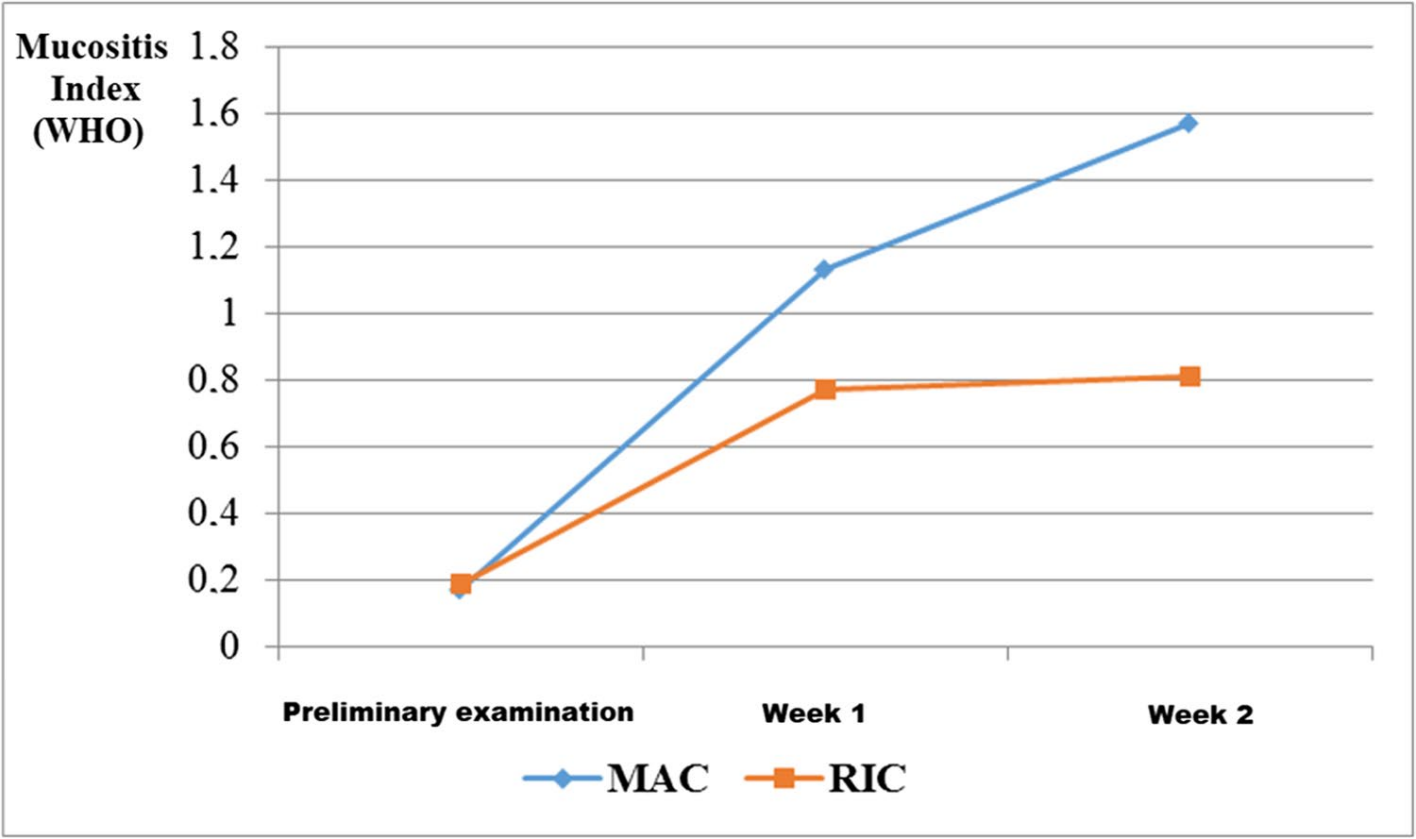
The mean value of the mucositis index according to the WHO criterion as determined from the three examinations for the two conditioning treatments (MAC, myeloablative chemotherapy; RIC, reduced intensity chemotherapy)

**Fig. 3:**
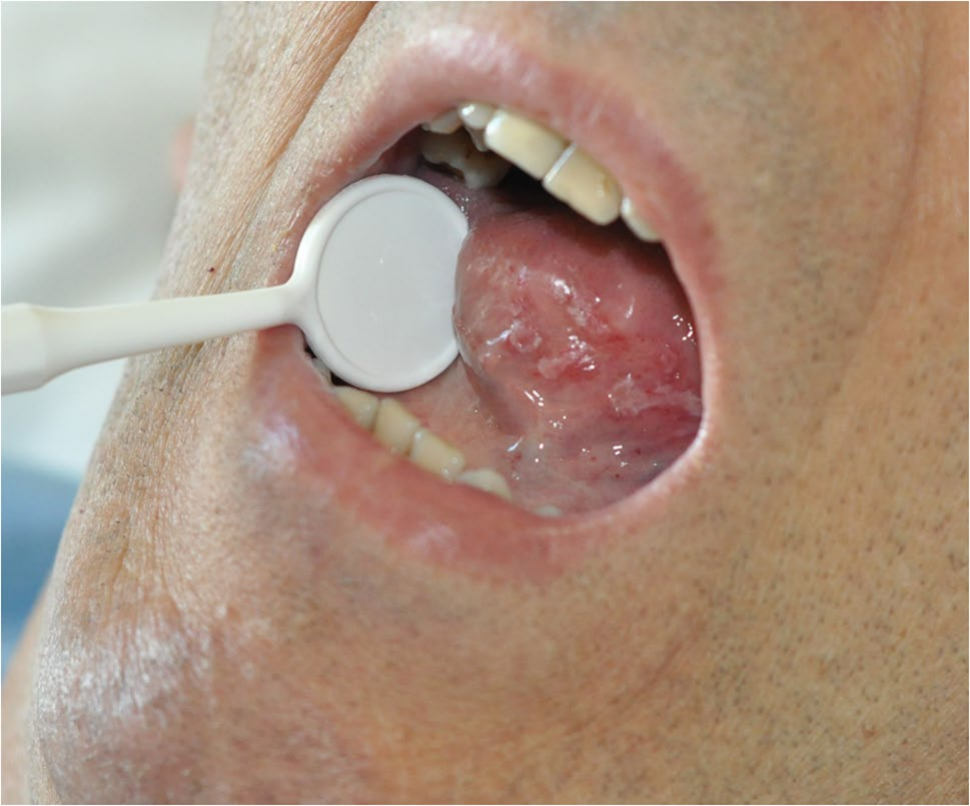
Mucositis stage 2°on the ventral surface of the tongue

**Fig. 4:**
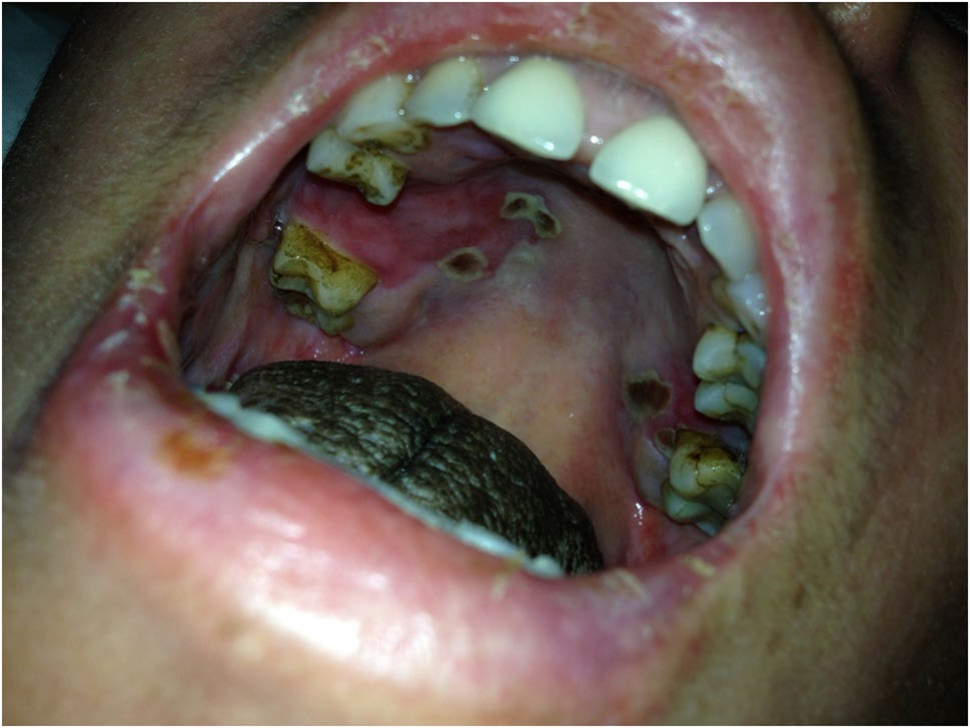
Mucositis stage 3° on the palatal, gingival, and labial oral mucosa accompanied by black hairy tongue

The highest mean value of the mucositis index 1.59, according to the WHO criterion, was recorded in the second week in the MAC group, while in the RIC group during the same period, it was found to be 0.8. In the RIC group, there was a slight increase in the index value in the second week compared to the first week, while in the MAC group, this level increased significantly during the examination following transplantation, compared to the preliminary examination. In both post-transplant studies, the severity of mucositis increased from that found during the initial examination but was greater in the MAC group compared to the RIC group (Figs. 2, 3, 4).

Three of the patients in the whole study population required parenteral nutrition during the therapy; however, oral mucositis was not the only reason for inducing this nutritional regimen. Two of these patients concomitantly developed a neutropenic enterocolitis. During the first week of the conditioning, parenteral nutrition was introduced in one patient from the MAC group and in one patient from the RIC group, while in the second week, one more patient from the MAC group qualified for this type of the nutrition. Type 4° mucositis was observed in all of these patients.

## Discussion

Mucositis is most common in patients undergoing intensive chemotherapy prior the stem cells transplantation. According to several authors, the frequency of those lesions ranges between 70 and 100% [18–21]. The Darczuk study examined a group of patients with several types of progressive, malignant neoplasms of the blood-forming organs, and mucositis was observed in 83% of patients in the first week post-transplantation and in 92% of patients in the second post-transplantation week [21]. Most of the reports on the frequency of oral mucositis in patients treated with allo-HSCT describe heterogeneous populations with various types of neoplasms. In our own study, where we focused on selected group of AML subjects, mucositis in stages 1–4 according to the WHO criterion was found in 66% of all patients in the first week and in 74% of patients in the second week post-transplantation.

According to various authors, symptoms of mucositis develop in 3–8 days after the commencement of chemotherapy [22–24]. The duration of lesions is variable — according to Borgman et al., the average duration is 8 days, while according to Borowski et al., the lesions last approximately 11 days, despite very meticulous oral hygienic procedures maintained by the patients [19, 20]. Krasuska-Sławinska et al. reported that the most severe mucositis developed during the 9th–10th day of therapy, while according to Olszewska, it usually occurs between the 7th and 14th day of treatment [24, 25]. Sonis observed that the most advanced stage of mucositis occurred 3–4 days before the peak of neutropenia, which is in 14th day after the beginning of chemotherapy [26, 27]. The results of several studies have shown that the intensity of the oral lesions depends on the level of myelosuppression, and the most advanced inflammatory lesions develop during the period of the highest reduction in the neutrophils count [28, 29]. In our own study on AML patients, oral mucositis was observed in most of the patients in the first week after the transplantation (7th–14th day from the beginning of the conditioning). The highest number of patients with oral symptoms was detected between 15 and 21th day from the start of cytotoxic therapy. Oral ulcerations induced by oral hygienic procedures and mastication may become secondarily infected by oral bacteria, mainly Gram-negative, which causes local tissue damage by releasing endotoxins to the oral epithelium. If the lesions are not secondarily infected, they heal spontaneously. According to Bendyk-Szeffer et al., the oral mucosa returns to its normal physiologic condition within 21 days from the termination of cytotoxic treatment [30].

In recent years, the use of reduced intensity conditioning (RIC) has been increasing. This therapy is suitable for elderly people and in patients with several comorbidities. The term RIC covers a wide spectrum of protocols, e.g., combinations of several cytostatic drugs and total body irradiation (TBI) in various doses. The protocols vary in the intensity of anticancer and myelosuppressive effects. A lower intensity may result in a lower risk of fatal complications related to the therapy, and also a lower risk of oral mucositis. However, at the same time, the risk of disease recurrence is higher. Current efforts are directed towards the development of conditioning protocols in order to maintain a high myeloablative potential with a low toxicity at the same time [17, 31].

Chaundhry et al. evaluated the frequency and severity of mucositis in relation to the type of conditioning in 624 patients who underwent allo-HSCT during the period 1990–2014 [32]. The total frequency of oral lesions reached 86.5% in the RIC group and 73.2% in the MAC group, while the severe stages (2–4°) of mucositis were observed slightly more often in the MAC group than in the RIC group (79.7% vs. 71.5%). Although the MAC protocol is generally characterized by a higher toxicity, which may suggest an increased frequency and severity of mucositis in patients treated with this regimen, some authors reported that both the frequency and the severity of oral lesions was comparably high in both groups. This could be partially explained by the diversity in the patient population qualified for each type of the conditioning. The RIC patients were generally older and suffered several comorbidities. Chaundry et al. also emphasized that the definitions of the conditioning types are based on the doses of cytostatics used, but this does not reflect the differences in their pharmacokinetic properties [32].

Legert et al., who examined 171 post allo-HSCT patients (mean age: 50 years), observed a lower mean severity of mucositis (according to the WHO classification) in subjects after reduced intensity conditioning compared to myeloablative therapy. Recently, the severity of mucositis has also decreased in patients treated with RIC compared to those previously treated. According to the authors, that could be explained by the more rigorous medical care focused on dental complications introduced in recent years [17]. Ringden et al., who compared the toxicity of both therapies in AML patients, also observed a lower percentage of the most advanced mucositis stage (according to the WHO classification) in RIC group (1) compared to MAC (4) [33]. In our own study, the frequency of post-transplantation mucositis was significantly higher in patients who underwent myeloablative treatment. Oral lesions were found in 74% patients from the MAC group and in half of the patients from the RIC group (50%) during the first week after transplantation. In the following week, the percentage of patients with mucositis increased to 83% in the MAC group, while in the RIC group, it remained almost at the same level (53%). Our results also demonstrated a higher severity of mucositis after transplantation in MAC patients compared to RIC patients. The highest mean value of mucositis (according to the WHO classification) was observed during the second week of therapy in the MAC group, with an index of 1.59. Meanwhile, at a comparable period of therapy, in RIC patients, this value was estimated to be 0.8. The symptoms of the most severe inflammation with diffuse necrotic lesions (mucositis type 4°) were found in both groups in a low percentage of patients and without significant differences in the frequency relative to the type of conditioning. Post-transplant stage 3 mucositis was also observed in both the MAC and RIC groups; however, during the second week of the therapy, the frequency of this complication was significantly higher in the MAC group. In general, the severe type of mucositis (2–4°) was revealed in 28% of MAC patients during the first examination and in 52% of MAC patients during the second examination, while in the RIC group, it was found in 15% and 19% of patients, respectively. In contrast to the results presented by Chaundhry et al., where severe mucositis after allo-HSCT was observed in 79.7% of MAC patients and in 71.5% of the RIC patients, these values were much lower. This may be due to the less toxic conditioning protocols introduced in recent years and more advanced prophylactic regimens focused on oral inflammatory complications [32].

The frequency of 0° mucositis in the MAC group decreased significantly in the subsequent examinations after transplantation, compared to the first examination. Meanwhile, the frequency of mucositis type 1°, 2°, and 3° increased significantly as revealed in both post-transplantation examinations when compared to the results from the examination prior to the transplantation.

## Conclusions

The results of our study indicate that the RIC therapy induces mucositis less frequently and with a lower intensity post-transplantation compared to myeloablative therapy, which is due to lower toxicity of RIC compared to MAC.

These effects significantly decrease the patients’ quality of life during the transplantation and may prematurely terminate the treatment. Considering the continuing growth in the number of transplantations performed on AML patients, further investigations of oral mucositis are required.

## Data Availability

The data that support the findings of this study are available from the corresponding author upon reasonable request.
